# Altered Satellite Cell Responsiveness and Denervation Implicated in Progression of Rotator-Cuff Injury

**DOI:** 10.1371/journal.pone.0162494

**Published:** 2016-09-26

**Authors:** Deanna Gigliotti, Jeff R. S. Leiter, Peter B. MacDonald, Jason Peeler, Judy E. Anderson

**Affiliations:** 1 Department of Biological Sciences, Faculty of Science, University of Manitoba, Winnipeg, MB, Canada; 2 Pan Am Clinic, Winnipeg, MB, Canada; 3 Department of Human Anatomy and Cell Science, College of Medicine, Faculty of Health Sciences, University of Manitoba, Winnipeg, MB, Canada; 4 Department of Surgery, University of Manitoba, and Pan Am Clinic, Winnipeg, Manitoba, Canada; University of Minnesota Medical Center, UNITED STATES

## Abstract

**Background:**

Rotator-cuff injury (RCI) is common and painful; even after surgery, joint stability and function may not recover. Relative contributions to atrophy from disuse, fibrosis, denervation, and satellite-cell responsiveness to activating stimuli are not known.

**Methods and Findings:**

Potential contributions of denervation and disrupted satellite cell responses to growth signals were examined in supraspinatus (SS) and control (ipsilateral deltoid) muscles biopsied from participants with RCI (N = 27). Biopsies were prepared for explant culture (to study satellite cell activity), immunostained to localize Pax7, BrdU, and Semaphorin 3A in satellite cells, sectioning to study blood vessel density, and western blotting to measure the fetal (γ) subunit of acetylcholine receptor (γ-AchR). Principal component analysis (PCA) for 35 parameters extracted components identified variables that contributed most to variability in the dataset. γ-AchR was higher in SS than control, indicating denervation. Satellite cells in SS had a low baseline level of activity (Pax7+ cells labelled in S-phase) versus control; only satellite cells in SS showed increased proliferative activity after nitric oxide-donor treatment. Interestingly, satellite cell localization of Semaphorin 3A, a neuro-chemorepellent, was greater in SS (consistent with fiber denervation) than control muscle at baseline. PCAs extracted components including fiber atrophy, satellite cell activity, fibrosis, atrogin-1, smoking status, vascular density, γAchR, and the time between symptoms and surgery. Use of deltoid as a control for SS was supported by PCA findings since “muscle” was not extracted as a variable in the first two principal components. SS muscle in RCI is therefore atrophic, denervated, and fibrotic, and has satellite cells that respond to activating stimuli.

**Conclusions:**

Since SS satellite cells can be activated in culture, a NO-donor drug combined with stretching could promote muscle growth and improve functional outcome after RCI. PCAs suggest indices including satellite cell responsiveness, atrogin-1, atrophy, and innervation may predict surgical outcome.

## Introduction

The rotator cuff muscle complex of the shoulder is comprised of 4 distinct muscles (supraspinatus, infraspinatus, teres minor and subscapularis) and controls shoulder movements (internal and external rotation) essential for performing normal activities of daily living such as eating, self-grooming, and lifting objects. Rotator cuff injury (RCI) to the supraspinatus muscle (SS), causes intense shoulder pain and weakness [1] through tendon tear and/or impingement, suprascapular nerve injury [2, 3], and inflammation [3]. Damage can be chronic and age-related, from repetitive strain or prolonged use, or acute and related to sudden, high-stress motion or impact [1, 4–6]. It is not understood why surgical repair of the SS tendon after RCI frequently fails to restore full joint function and stability. Failure rate ranges from 30–94% [7], which is reported to relate to adverse changes in muscle, including fatty infiltration, fibrosis and/or denervation [8, 9]. The present study reports further findings on the biology of muscle stem cells and innervation status from phase 2 of an ongoing study of SS and ipsilateral control deltoid muscles, biopsied during arthroscopic surgery.

Satellite cells (SCs) are mitotically quiescent muscle stem cells located between the basal lamina and the muscle membrane [10, 11], and need to be activated before they can proliferate and contribute to muscle repair. With muscle activity, SCs are activated in a signalling cascade initiated by calcium influx following injury or mechanical strain on the sarcolemma, mediated by nitric oxide (NO). Upon activation, SCs enter G1, migrate to the area of damage, and proliferate [12–15]. Quiescent SCs in adult muscle express the transcription factor, Pax7; its expression either decreases as myoblasts differentiate and express the muscle regulatory factor, myogenin or is maintained while the cells return to quiescence as the self-renewing fraction of stem cells [16]. Drugs such as isosorbide dinitrate (ISDN) release NO and can stimulate SC activation *in vivo* in dystrophic [17, 18] and old wild-type mice [19] and *in vitro* in cultures of muscle [20] and fibers [21]. Our first report on RCI-affected SS muscle explants showed ISDN activated SCs in SS but not the control ipsilateral deltoid muscle [22]. The impact of this difference to developing new treatment approaches is important enough to make confirmation of the results essential.

While the relationship between SC capacity for activation and the innervation status of SS after RCI is not understood, recent research has implicated SC signaling in the process of muscle reinnervation. SC have potential to influence axon growth and the reappearance of NMJs by their secretion of semaphorin 3A (Sema3A) [23, 24]. Sema3A is a neural chemorepellent that is thought to coordinate the reconnection of motor axons with a differentiating fiber in a regenerating muscle [25]. The current model is that a decline in Sema3A would allow motor neurites to re-contact repaired fibers and possibly influence the fiber-type patterning of muscle [24], although this has not been investigated in human or pathological muscle.

Acetylcholine receptors (AchRs) cluster as pentamers at neuromuscular junctions [26] in a multi-step process stabilized by rapsyn [27] and agrin [28]. The AchR complex includes 2 alpha, 1 beta, and 1 delta subunit, plus a fifth subunit that varies with maturation and innervation. Fetal muscle and denervated muscle express the gamma (γ) subunit, while active, adult, innervated muscle expresses the epsilon (ε) subunit [29] and little γ-subunit [27, 30–32]. Denervation atrophy and weakness (such as occurs in aging) can lead to significant fibrosis and fatty infiltration in human muscle [33–38] and reduce treatment efficacy [39]. Longer-term denervation impairs the potential for reinnervation [33]. At least in mice, denervation combined with vascular injury delays and reduces the success of muscle repair [40].

Study of biopsies from 13 participants with RCI showed a trend toward SS denervation, in that the γ:ε ratio of AchR subunits tended to be higher in SS than control muscle at the time of arthroscopic repair [22]. Intriguingly, in the same biopsies, only the SCs in SS explants were activated by treatment with ISDN in culture; SCs in deltoid explants did not respond to ISDN. Further study of these phase-1 samples together with a second phase-2 cohort showed SS had fiber atrophy, fibrosis, reduced vascular density, and a reduced proportion of type-1 slow fibers expressing MyHC1 compared to control deltoid [22]. Here the AchR subunit patterning and innervation status were further investigated in relation to the impact of ISDN to activate SCs into S-phase and SC production of Sema3A. Principal component analysis of features of demographics, life history, MRI, tissue histology and protein-expression was conducted on data from over 40 variables to identify particular features that explain a significant portion of the variation in the dataset. Those variables are expected to be important in understanding muscle status in RCI and interpreting the outcome of new interventions designed to treat the condition.

## Methods

Tissues were collected after informed written consent (record kept on file at Pan Am Clinic, Winnipeg, Manitoba, Canada), under a protocol that included a template consent form and was approved by the Biomedical Research Ethics Board at the University of Manitoba (B2010:074, extended in 2013–14). Participants had clinical evidence of a rotator cuff tear and had failed conservative management. Inclusion-exclusion criteria, participant demographics, and findings from initial investigations on phase-1 participants (N = 13) were reported previously [22]. The second cohort (phase-2 participants) was the next 15 consecutive subjects (1 without a SS tear, was excluded at the time of surgery), and partial findings on phase-2 participants are reported elsewhere [41]. The study dataset represents a total of N = 27 participants (phase-1 plus phase-2). Participants (20 males, 7 females) were 56.6 ± 1.5 years-of-age (range 45–66 years) with right (N = 16) or left (N = 11) shoulder affected by traumatic (N = 19) or non-traumatic (N = 8) injury resulting in full-thickness (N = 25) or partial-thickness (N = 2) tears of SS. The cohort (8 smokers and 19 non-smokers) had a body-mass index of 28.4 ± 4.3 (height 1.7 ± 0.11m; weight 86.4 ± 17.8 kg), and the mean time from onset to surgery was 72.5 weeks (range 12–520 weeks).

Control (deltoid) and SS biopsy samples were coded to reduce bias in all assays; data were decoded just prior to statistical analysis. Tissues were frozen for cryosectioning and immunostaining, cultured as explants to study SC activation and Sema3A localization and their respective responses to NO or processed for protein extraction and western blotting.

### Explant cultures: SC activation and Sema3A responsiveness

Samples of SS and control muscles were cultured in medium containing bromodeoxyuridine (BrdU, 0.65 μM, Sigma Aldrich Co., St. Louis, MO), with or without ISDN (236.14μg/L) for 40 hours exactly as reported, before fixation and cryosectioning [22].

One set of slides was processed for double immunofluorescence to detect SCs positive for both BrdU and Pax7, using rabbit anti-Pax7 (1:150; ab34360, AbCam, Cambridge, UK) and mouse anti-BrdU (1:200; 11–170376001, Roche, Basel, Switzerland), then secondary antibodies goat anti-rabbit Dylight-488 (1:200) and goat anti-mouse Dylight-649 (1:200), respectively. Sections were coded, scanned, and photographed systematically from 20 fields using a 63X oil immersion lens on a Zeiss ApoTome. Pax7+ SCs were counted as reported [22], as activated (BrdU+) or not (BrdU-) and the proportion of activated SCs was calculated.

A second set of slides was processed to detect Sema3A using rabbit anti-Sema3A (1:300; ab23393-50, AbCam, Cambridge, MA), biotin-conjugated goat anti-rabbit (1:200; 111-067-003, Jackson ImmunoResearch, West Grove PA), horseradish peroxidase (HRP)-conjugated streptavidin (1:500; Vector Labs, Burlingame, CA), and the 3,3’-diaminobenzidine detection system [22, 25]. Sections were viewed and photographed on a BH-2 Olympus microscope using a UC50 digital camera (Olympus, Tokyo, Japan) with imaging software (Olympus Soft Imaging Solutions). A maximum of 20 non-overlapping images per section were captured under oil immersion (X1000). The areal density of staining for Sema3A (mean optical intensity/stained area) was measured in all Sema3A+ cells in the satellite position on fibers, interpreted as SCs, using ImageJ software (NIH, Bethesda, MD), and data were analyzed in Microsoft Excel 2010.

### Protein expression studies by western blot

Western blotting followed standard lab protocols [19, 42] using the Santa Cruz luminol detection system for HRP with chemiluminescence detection to visualize and quantify proteins of interest. Proteins were detected with the following antibodies and reagents: goat anti-ε-AchR subunit (1:150; sc1454, Santa Cruz, Dallas, TX) and HRP-conjugated goat anti-mouse (1:5000; A2304, Sigma Aldrich); rabbit anti-γ-AchR subunit (1:250; sc-13998, Santa Cruz), biotin-conjugated goat anti-rabbit (1:200; 111-067-003, Jackson ImmunoResearch) and HRP-conjugated streptavidin (1:500; Vector Labs); and mouse anti-β actin (1:1500; sc81178, Santa Cruz) and HRP-conjugated goat anti-mouse (1:5000; A2304, Sigma Aldrich). After detection and imaging for one antigen, membranes were quenched and re-probed to detect a second antigen [42]. Membranes were incubated in luminol (1 min.), covered in plastic film in the VersaDoc (BioRad Inc., Hercules, CA), and imaged using the QuantityOne program over 600 seconds with increasing exposure at 100s increments. ImageOne software was used to measure band density, which was tabulated in Excel 2010 for analysis. Protein levels were expressed relative to a loading control, β-actin, and compared between SS and control muscles.

### Statistics and Principal Component Analysis

Data were analyzed using paired Student’s t-tests or 2-way analysis of variance, as appropriate to compare differences between SS and control muscles. A probability of p<0.05 was used to indicate significance.

Principal component analysis (PCA) was performed to identify variables that contributed the most, to the variability in the dataset. PCA summarizes the information in a dataset with a large number of variables, by identifying a small number of new components; it thus accounts for most of the variation in the dataset by using fewer factors while retaining correlations among the variables. Graphical representation of significant results (termed “loading plots” or biplots) is useful in selecting those components that preserve the most important information in the original dataset. The new components, called principal components (PCs), are formed by groups of variables in the original dataset. PCs are extracted by linear transformation of data into eigenvectors of covariance or correlation matrices, and graphically represent the variability in the original dataset. The first new variable, termed PC1, is the vector or component that accounts for the most variance in the data; PCs 2 and beyond, account for the next highest levels of variation in declining order. PCA assumes that: variables are linearly related, the mean and variance of the data describe the distribution, and the largest variances are the most important [43, 44]. Data were evaluated in relation to these assumptions through graphical checks for outliers and any obvious non-linear relationship among variables. Transformations, including log or square root functions, were used if needed to improve the linearity of the data, and increase the ability to extract eigenvectors. All data for phase-1 and phase-2 participants were compiled for the PCA using the programming language, “R”.

## Results

### AchR subunits

**Fig 1:**
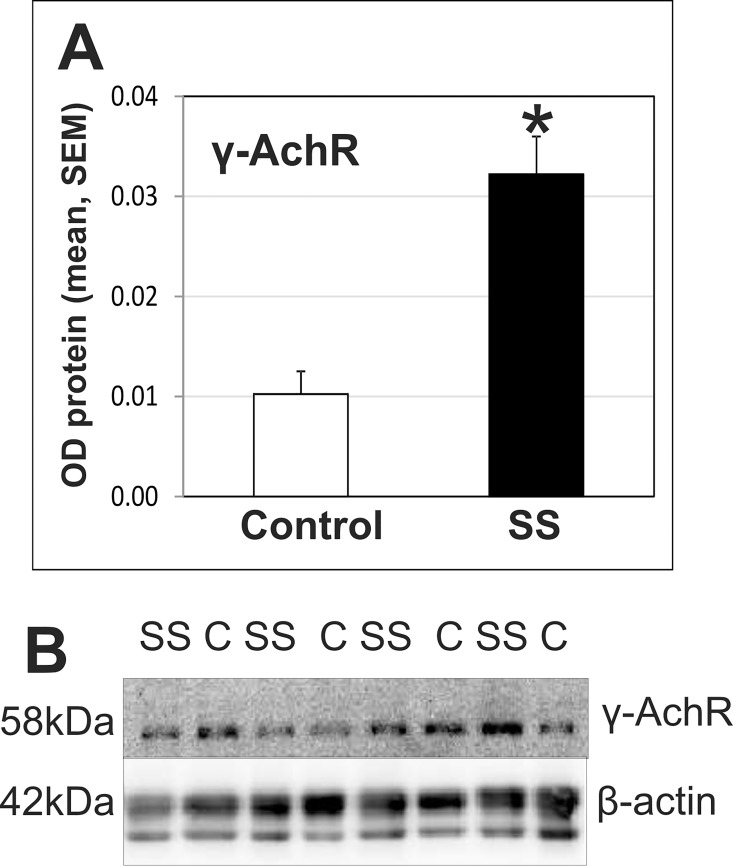
Changes in the level of the γ-AchR subunit. A. Graph of the optical density (OD) of bands from western blots probed for the γ-AchR subunit (relative to β-actin) in SS compared to control muscle. The amount of the γ-AchR subunit protein, typically expressed in denervated and fetal muscle, was assessed in protein extracts prepared from homogenized SS and control deltoid muscle (* indicates significant difference, p<<0.001, N = 19 paired samples). B. A representative western blot prepared from protein extracts loaded into lanes for control (C) and Supraspinatus (SS) muscles from different participants, probed to detect the γ-AchR subunit and then re-probed to detect β-actin (as a loading control).

The ε subunit in AchRs reverts to the γ subunits upon denervation. Here, the expression of the two AchR subunit proteins was quantified by western blot. Expression of the γ-AchR subunit (standardized to β-actin) was significantly higher in SS than in the control muscle (p<<0.001, N = 19), implicating some impact of SS denervation at the time of the biopsy (Fig 1). The γ:ε ratio of AchR subunits, which can be used as an indicator of innervation status in a muscle [22], also tended to be higher than control (p = 0.09, N = 20).

### SC responsiveness

#### Satellite Cell Activation

**Fig 2:**
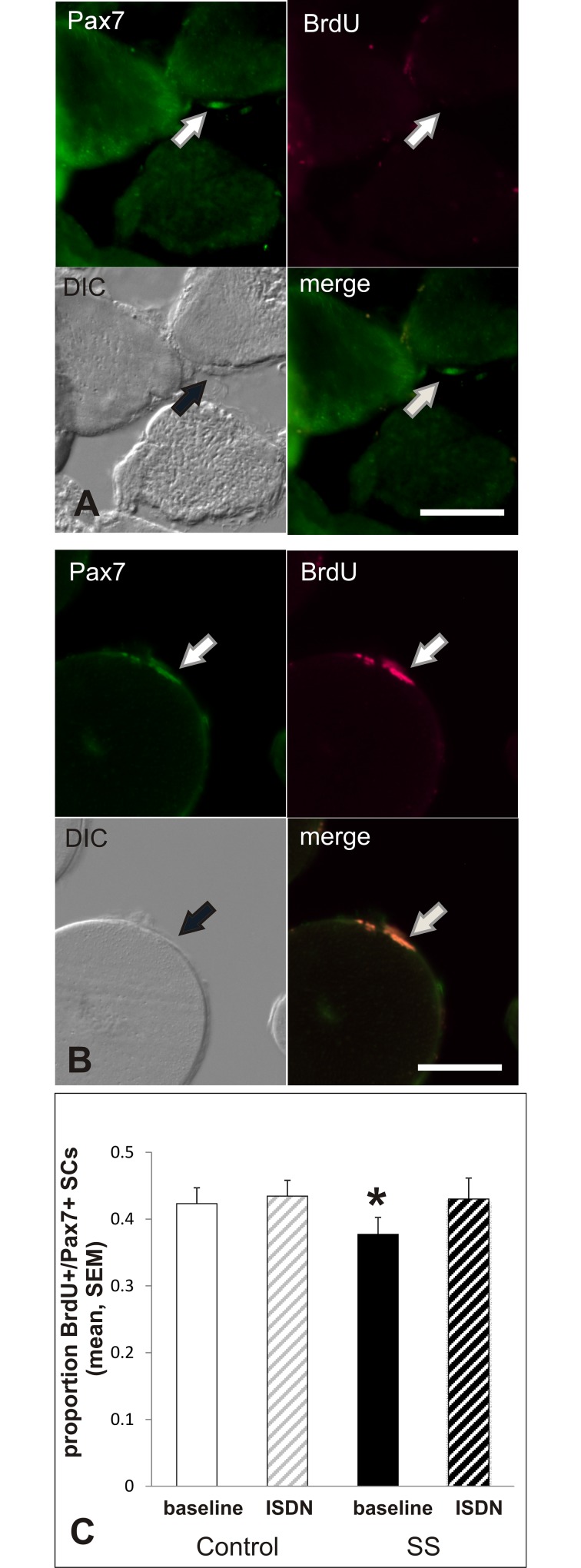
SC activation in response to ISDN in explant cultures. A and B. Micrographs of immunofluorescence staining for BrdU and Pax7. Images are as labeled: DIC for orientation to the SC; Cy5 channel used to visualize anti-BrdU staining; GFP channel used to visualize anti-Pax7 staining; and a merge of Cy5 and GFP to show any overlap. A. shows staining of a Pax7+/BrdU- cell. B shows a Pax7+/BrdU+ SC where merge of Cy5 and GFP is yellow. C. Graph of the proportion (mean, SEM) of active BrdU+/Pax7+ SCs of the total number of Pax7+ SCs observed in sections of control or supraspinatus (SS) muscles, after culturing for 40 hours in the presence of BrdU, with isosorbide dinitrate (ISDN) or at baseline (no treatment). At baseline, SS had a lower proportion of activated SCs than control muscle. ISDN increased the number of BrdU+ SCs in SS but not control muscle. Asterisk (*) indicates significant difference from untreated SS (p = 0.01, N = *27*) from a total of 7661 Pax7+ satellite cells.

The initial phase-1 study of SC responsiveness to activation by ISDN in culture found that SS had a lower baseline level of active proliferation (BrdU+/Pax7+) SCs than control muscle (p = 0.03, N = 13). In that study, ISDN increased the proportion of BrdU+ SCs only in SS, and the level of activity after ISDN increased up to the baseline level found in SCs of control muscle. The current, independent-repeat experiment on phase-2 samples gave identical results (p = 0.03, N = 14) from data on a total of 5016 Pax7+ satellite cells photographed and counted from the four groups of muscles (Control baseline: N = 117; Control ISDN: N = 1171; SS baseline: N = 1371; SS ISDN: N = 1295). Fig 2 shows results from the full dataset; the level of BrdU+/Pax7+ SCs in baseline SS cultures was significantly lower than the level in all other groups (p = 0.016, N = 27). The data represented in Fig 2 include counts of the following number of Pax7+ satellite cells (N = 27 participants): Control baseline: N = 1915; Control ISDN: N = 1862; SS baseline: N = 1946; and SS ISDN: N = 1938.

#### Sema3A localization

**Fig 3:**
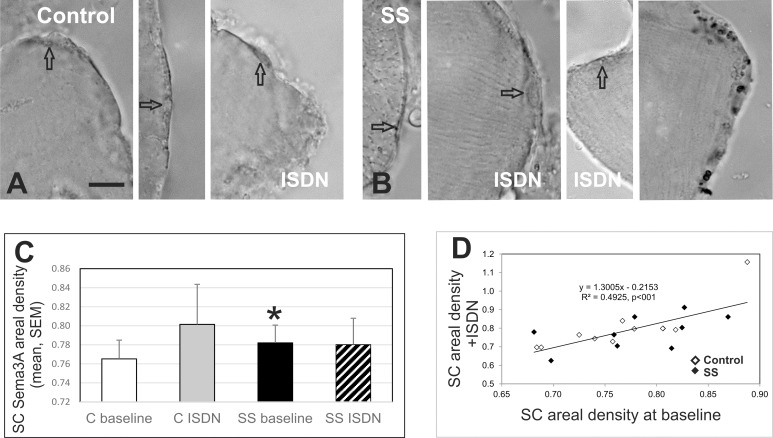
Localization of Sema3A protein. Representative micrographs of Sema3A staining in presumptive SCs, located in the satellite position on fibers (arrow indicates nucleus) in control and Supraspinatus (SS) muscle explant cultures at baseline (without ISDN treatment) or with ISDN (as labeled) [a subset of n = 10 participants from the full dataset shown in Fig 2]. Bar = 20 μm. A. Cells in control muscle ranged from large and activated (left panel) to small and attenuated (middle panel), with low to moderate intensity staining in the cytoplasm. Sema3A staining of an attenuated cell in the satellite position on fibers in ISDN-treated control muscle have dark staining for Sema3A. B. Most SCs in SS muscle at baseline had attenuated moderately-stained cytoplasm (left panel), and were larger with low to moderate intensity staining after ISDN treatment. Right-most panel shows dark Sema3A staining in a Schwann cell close to a NMJ. C. Graph of areal density of Sema3A stain (mean, SEM) in control (C) and supraspinatus (SS) muscle at baseline or after treatment with ISDN. Sema3A staining was higher in SS at baseline than in control muscle at baseline. Asterisk (*) indicates significant difference from control muscle (p = 0.04). D. Graph illustrating the significant correlation of SC areal density after activation by ISDN (y-axis) with that at baseline in the same muscle (control, open diamonds, SS, black diamonds); R2 = 0.4925, p<0.001, N = 10.

The neural chemorepellent, Sema3A, secreted by activated SCs, is implicated in muscle re-innervation [23–25, 45]. Observations of staining for Sema3A in muscles cultured with or without ISDN treatment (Fig 3A and 3B) showed that the area of cytoplasmic staining of cells in the satellite position (interpreted as SCs) varied in sections, with generally larger cells stained in control muscle compared to the more attenuated cells stained in SS muscle at baseline. This observation is consistent with the finding that SC in SS muscle at baseline had a lower level of activation than in control muscle at baseline (Fig 2). In muscles treated with the SC-activating NO-donor, ISDN, there was a subjective increase in the size of these cells in SS but not control muscle. Staining varied from low to high, in both muscles; the stained area of cytoplasm varied from a narrow attenuated region in thin cells to larger areas of cytoplasm in the cells that were apparently more activated as they contained larger nuclei, as previously reported [15]. Sema3A staining appeared quite dense in the cytoplasm of Schwann cells observed in proximity to neuromuscular junctions (Fig 3B, right image); Schwann cells are another source of this secreted protein [46].

The areal density of SC Sema3A staining (mean intensity/SC area) was measured in micrographs of all cells in the satellite position (interpreted as SCs, typically 12-18/section) identified by a nucleus and clear delineation of SC cytoplasm from the adjacent fiber, observed at 1000X. The baseline density of Sema3A staining in these cells of SS was higher than in control muscle (p = 0.04, N = 10) (Fig 3C). While culture in ISDN for 40 hours did not increase this measure of Sema3A protein localization in either muscle, cells in the satellite position on control fibers tended to have a higher Sema3A areal density after treatment (p = 0.11, N = 10). Sema3A areal density was also highly correlated in the two muscles of each participant (R^2^ = 0.81, F_1_ = 34.6, p<0.001, N = 10, not shown). Sema3A areal density after culture with ISDN was also correlated to the density at baseline in the same muscle (R^2^ = 0.49, F_1_ = 17.5, p<0.001, N = 10, Fig 3D).

### Principal Component Analysis (PCA)

**Fig 4:**
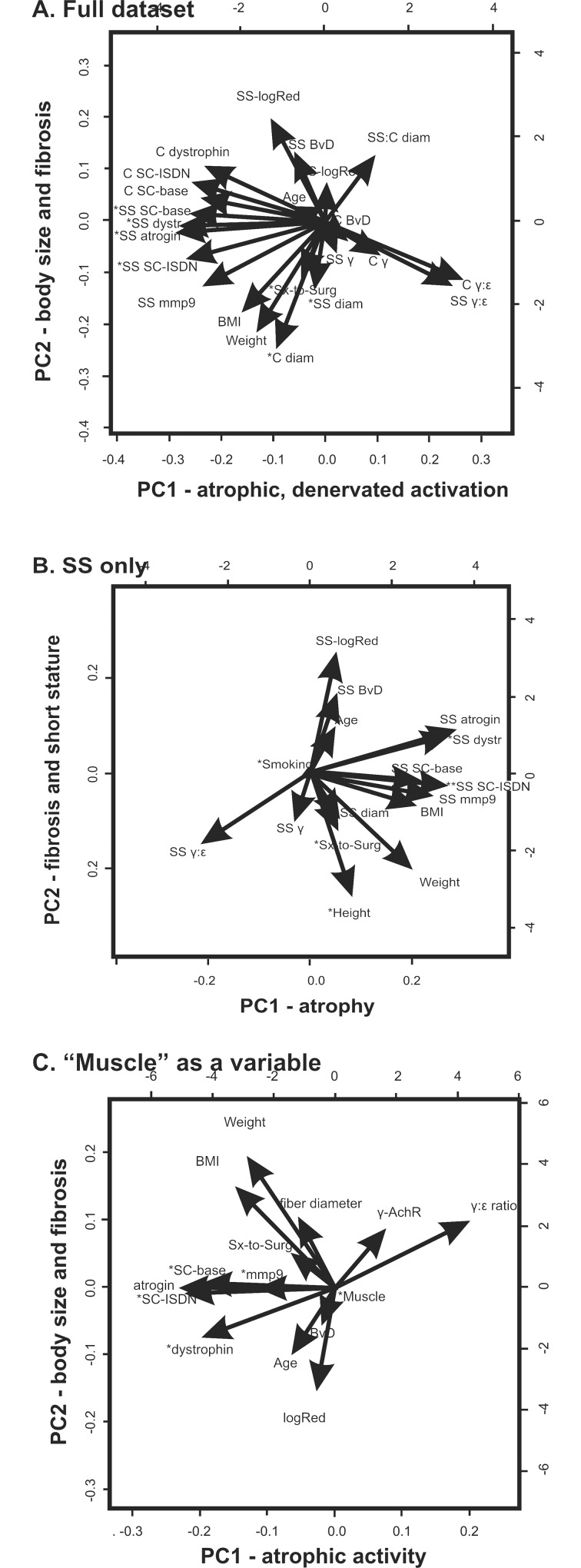
Principal component analysis (PCA) biplots. Biplots show the correlation vectors representing the projection (in 2 dimensions) of loading for each of the variables (for 27 participants) included in the particular PCA. Vectors project onto the 3 axes (dimensions) of the principal components (PCs) 1 (x-axis), 2 (y-axis), and 3 (z-axis, positive is upward, perpendicular to the page). A. PCA-1, on the full dataset (N = 23 variables). B. PCA-2, Supraspinatus variables only (N = 16). C. PCA-3, variables of interest from previous comparisons, including “muscle” as a variable (N = 15 variables). Variables that loaded onto PC3 (as shown in Tables 1–3) are indicated with an asterisk (*) to emphasize that the correlation vector projects upward, perpendicular to PC1 and PC2 axes. Variable labels on the vectors are abbreviated as follows: C (control muscle), SS (Supraspinatus muscle), BMI (body mass index), Sx-to-Surg (weeks from symptom onset to surgery), diam (fiber diameter), SS:C diam (ratio of fiber diameter in SS/C), SC-base (baseline SC activation in explant cultures), SC-ISDN (SC activation in explants cultured with ISDN), dystr (expression of dystrophin protein relative to β-actin), mmp9 (level of matrix metalloproteinase 9 protein relative to β-actin), logRed (log-transformation of average maximum Sirius Red staining), γ:ε (γ:ε ratio of AchR subunits), γ (expression of the γ-subunit of AchR protein relative to β-actin), and BvD (vascular density).

PCA was used to reduce the dimensionality of the multivariate dataset for the current study, in which a total of 35 parameters were assessed (whether or not individual statistical analyses, above, were significant). The large dataset was reduced to data in three dimensions (principal components, PCs) which helps visualize statistical findings from an analysis of many variables together. Use of PCA can therefore be preferable to examining many variables using multiple pair-wise correlations. Fig 4 shows plots of the results of PCAs that were run on the data in the current RCI study, following standard protocol [43]. Interpretation of these plots considers the vectors (direction and length) as representing the coefficients of each of the variables with the PC axes, and the numbers are located at points representing the loading (or correlation) scores of individual observations (participants or muscles) on the PCs in the plot. To be considered important in defining the PC, the absolute value of a loading coefficient for variable was set at ≥ 0.3 (PCA-1) or ≥ 0.35 (PCA-2, PCA-3).

**Table 1:** Principal Component Analysis (PCA) table of the full dataset on N = 23 variables, showing correlation coefficients for variables loaded on the 3 PCs extracted from the analysis of data from 27 participants. In the row for each variable, numbers indicate the strength of correlation of that variable with the eigenvector of each PC. When the absolute value of correlation coefficients was ≥ 0.3, they were considered important (bold font) in defining the PC. Variables loaded on PCs 1–3 below, appear in the PCA plot (Fig 4A) with an asterisk (*) to indicate they also project upward (since PC3 is perpendicular to axes for PCs 1 and 2).

Variable	PC1	PC2	PC3*
Weight	-0.152	**-0.375**	
Body mass index	-0.185	**-0.309**	
Weeks from symptoms to surgery		-0.193	**-0.398**
SS fiber diameter		-0.232	**0.467**
Control fiber diameter	-0.108	**-0.431**	**0.325**
Activated SCs in SS with ISDN	**-0.309**	-0.125	-0.195
Activated SCs in SS at baseline	-0.275		**-0.322**
Control γ:ε AchR ratio	**0.302**	-0.197	
SS atrogin-1	**-0.331**		0.185
SS dystrophin	**-0.331**		0.106
SS fibrosis (avg. max. Red OD)	0.121	**0.349**	
**% variance explained**	**30%**	**12.6%**	**9.8%**

**Table 2:** Table of PCA-2 on data only from the Supraspinatus (SS) muscle, using N = 16 variables, showing correlation coefficients for variables loaded on the 3 PCs extracted from the analysis of data from 27 participants. In the row for each variable, numbers indicate the strength of correlation of that variable with the eigenvector of each PC. When the absolute value of correlation coefficients was ≥ 0.35, they were considered important (bold font) in defining the PC. Variables loaded on the PCs 1–3, appear in the PCA plot (Fig 4B) with an asterisk (*) to indicate they also project upward.

Variable	PC1	PC2	PC3*
Height	0.118	**-0.478**	0.236
Smoking status			**0.527**
Weeks from symptoms to surgery		-0.223	**-0.486**
Activated SCs in SS with ISDN	**0.384**		-0.239
SS atrogin-1	**0.406**	0.170	
SS dystrophin	**0.394**	0.156	0.157
SS fibrosis (avg. max. Red OD)	0.121	**0.473**	
SS vascular density		0.314	**0.359**
**% variance explained**	**28.3%**	**16.4%**	**12.8%**

**Table 3:** Table of PCA-3 using 15 variables of interest, including “muscle” (i.e., SS or control), showing correlation coefficients for variables loaded on the 3 PCs extracted from the analysis of data from 27 participants. In the row for each variable, numbers indicate the strength of correlation of that variable with the eigenvector of each PC. When the absolute value of correlation coefficients was ≥ 0.35, they were considered important (bold font) in defining the PC. Variables loaded on PCs 1–3, appear in the PCA plot (Fig 4C) with an asterisk (*) to indicate they also project upward.

Variable	PC1	PC2	PC3*
Muscle			**0.561**
Weight	-0.246	**0.553**	
Body mass index	-0.280	**0.406**	
Activated SCs with ISDN	**-0.380**		-0.200
Activated SCs at baseline	**0.363**		-0.187
γ:ε AchR ratio	**-0.379**	0.267	
Atrogin-1	**-0.435**		
Dystrophin	**-0.374**	-0.208	-0.150
MMP9	-0.209		0.613
SS fibrosis (avg. max. Red OD)		**-0.418**	
**% variance explained**	**26.7%**	**12.6%**	**11.1%**

PCA-1 included all the variables available for both muscles (Table 1 and Fig 4A). PC1 was defined by the proportion of active (BrdU+/Pax7+) SCs in ISDN-treated SS explant cultures, the level of atrogin-1 in SS, the level of dystrophin in SS, and the γ:ε ratio of AchR subunits in control muscle. PC1 was named “atrophic, denervated activation” and accounted for 30% of the total variance in the dataset.

PC2 accounted for 12.6% of the total variance and included body weight, body mass index, fiber diameter in control muscle, and fibrosis in SS (average maximum-Red intensity by Sirius Red staining); PC2 was named “body size and fibrosis”.

PC3 included variables for the number of weeks between symptom onset and surgery, fiber diameter for SS and control muscles, and the proportion of active SCs in SS at baseline (without ISDN treatment in culture). PC3 accounted for 9.8% of the variation in the dataset, and was called “muscle status and time since injury”.

In PCA-1 on the full dataset, the three PCs together, accounted for over half of the total variance in the dataset. In a second PCA run on only data from the variables within the 3 PCs from PCA-1 (a subset of 11 variables including data for both muscles), the PCs explained 75% of the variation in the same combination of PCs (not shown).

PCA-2 was run only on data for the SS muscle and included variables from the MRI assessment which was not done on the control muscle. For PCA-2 (Table 2 and Fig 4B) the loading of a variable had to be >0.35 to be considered, PC1 represented the levels of atrogin-1 and dystrophin proteins and the proportion of active SCs in ISDN-treated samples in culture; PC1 accounted for 28.3% of the total variance in this dataset, and was called “atrophy”. PC2, called “fibrosis and short stature”, accounted for 16.4% of the total variance and represented participant height and fibrosis (average maximum-Red intensity). PC3 included the number of weeks between symptom onset and surgery, vascular density, and smoking status. PC3 was designated “time since injury and vascularity”, and accounted for 12.8% of the total variance. In PCA-2, PCs accounted for 57.5% of the total variance.

PCA-3 was run including “muscle” as a variable, and included those variables that were considered informative from the earlier (non-PCA) analyses (Table 3 and Fig 4C). PC1 represented the levels of atrogin-1 and dystrophin, the proportion of active SCs in baseline and ISDN-treated samples in culture, and the γ:ε ratio of AchR subunits; PC1 accounted for 26.7% of the total variance and was called “atrophic activity”. PC2 accounted for 12.6% of the total variance and included body weight, body mass index, and fibrosis, again designated “body size and fibrosis”. PC3 represented muscle (control or SS as a variable) and the level of matrix metalloproteinase (mmp9), and accounted for 11.1% of the total variance. PC3 was designated “muscle type and collagen degradation”. Together, the three PCs in PCA-3 accounted for 50.4% of the total variance in the data.

In these PCAs, each of the 3 PCs accounted for ~10% or more of the variation, and together explained over half the total variation in the respective datasets. Given the range observed in each of the variables in a dataset that included males and females with various medical interventions before surgery, and diverse histological, molecular, experimental, and clinical indicators, this explanation of variance is considerable. Two variables: the proportion of active SCs (BrdU+/Pax7+ SCs) in ISDN-treated explant cultures, and the quantification of fibrosis through Sirius Red staining of collagen, were always loaded onto (meaning correlated with) at least one PC in every PCA. The strongest correlations of variables were: SC activation in SS-IDSN, control fiber diameter, and SS fiber diameter in PCA-1 (all 23 variables); atrogin-1, height and smoking status in PCA-2 (SS muscle variables); and atrogin-1, weight, and MMP9 in PCA-3 (with “muscle” as a variable). It is interesting that MMP9 would show such a high correlation with PC3 in PCA-3 (loading of 0.613), despite there being no difference in the level of MMP9 between SS and control muscle.

## Discussion

Experiments reported here, on muscle innervation status and SC responsiveness, were part of a larger investigation of the cellular and molecular pathology of RCI muscle. Participants had a diagnosis of RCI, and failure of at least 6 months of non-surgical treatment, for both phase-1 [22] and phase-2 [41] of the study design. The first report on phase-1 participants revealed that atrophic SS muscle showed trends indicative of possible denervation compared to control muscle, and identified the use of ISDN in culture to activate SCs as a way of assessing differential SC responsiveness in the two muscles [22]. Initial studies on muscle from phase-2 participants showed SS muscle atrophy was accompanied by a decrease in the frequency of slow-type fibers expressing MyHC1 protein, a lower vascular density, and increases in atrogin-1, dystrophin, and VEGF proteins compared to the ipsilateral control muscle.

The current report extends the dataset on human SS and control muscles by investigating fibrosis, two modalities of SC responsiveness (activation and Sema3A localization) to the NO-donor ISDN, and the status of muscle innervation. The overarching hypothesis of this large study was that changes in innervation status (AchR-subunit composition), fibrosis (collagen deposition), SC activation, Sema3A localization to SCs, and changes in expression of other indicator proteins accompany the atrophy of SS in RCI. Results now identify that, compared to control muscle, SS had significant fibrosis, a higher level of γ-AchR and displayed SCs with less proliferative activity at baseline and greater responsiveness to activation by ISDN. SS also contained SCs with a higher level of Sema3A localization at baseline vs. control, which did not change with ISDN treatment. Findings implicate denervation in SS fiber atrophy, and show the important potential for SS muscle growth by treatment to promote SC activation (before and/or after tendon repair). In addition, PCA identified sets of key variables related to atrophy, protein degradation, the time between injury and surgery, vascular density, and especially SC activity after ISDN treatment and the level of fibrosis, as important in explaining variance in a large dataset from 27 participants. This is the first study of RCI muscle that specifically investigates the responsiveness of muscle satellite cells and the profile of denervation that also applies the power of PCA to interpret a wide range of assays. Insights into the condition of SS in RCI in this investigation of possible denervation identify the potential for pre-operative treatment targeted to promote SC activation, angiogenesis and/or re-innervation of SS muscle to improve surgical outcome of RC repair.

In this study, the ipsilateral deltoid muscle served as the control muscle, since the contralateral SS is often affected by RCI, sometimes asymptomatically. As well, the deltoid is reportedly not affected in patients with symptoms of RCI [1]. By contrast, a high prevalence of contralateral RC tears is reported in participants with medium or larger RC tears, investigated using ultrasonography [47], further constraining reliable use of the contralateral SS as a control muscle. An earlier study on muscle progenitor cell regenerative capacity in RC tears also used deltoid muscle as a non-RC control, to study arthroscopic findings in SS and infraspinatus RC muscles [22, 48]. Therefore, the current study was designed to biopsy ipsilateral deltoid as a comparison to the SS muscle of participants with RCI, and thereby control for variables such as age, life history, and activities such as smoking status, that would be important considerations in interpreting changes in muscle parameters and overall health [48].

It was interesting, and somewhat surprising that NO-donor treatment did not stimulate satellite cell activation in the control muscle in this study of muscle explant cultures from phase-2 participants or the initial study of muscle explants from phase-1 participants [22]. This is particularly the case, given the work from our lab on the role and impact of NO on satellite cells in wild-type and dystrophic mdx, and transgenic NOS-1 knockout mouse muscle *in vivo* during regeneration [15, 49], on muscle growth induced by exercise of 19-month-old mouse muscle [19, 50], on satellite cells resident on isolated muscle fibers from wild-type, dystrophic, and NOS-1 knockout mice and in muscle cultures studied in culture experiments with and without stretching [12, 20, 51–54]. However, as indicated by recent studies on zebrafish fibers in culture, there is a dose-response curve for NO-mediated activation by satellite cells in wild-type muscle [21], previously proposed as underlying the regulation of activation from quiescence [53, 55, 56]. However, the two sets of experiments on explant cultures from participants in phase-1 [22] and phase-2 (current report) of this study were identical and significant to the same probability (p<0.01). Thus the lack of a response to NO-donor treatment by satellite cells in the human control muscle in this clinical study is interpreted as indicating that satellite cells in the control muscle would be activated by higher-dose NO-donor treatment than in this study. Findings are also interpreted to indicate that satellite cells in the supraspinatus muscle that showed atrophy, fibrosis, and were at least partly denervated, were in essence “hyper-responsive” to NO-donor treatment, relative to control muscle. This relatively high responsiveness in SS or lack of response in control muscle, would be similar to the findings (initially counter-intuitive) of satellite cell responses to an activating stimulus (via NO, hepatocyte growth factor, or stretch) on muscle fibers from mdx mice [53, 55]. These ideas likely account for the present findings that satellite cells in atrophic SS muscle were activated at the dose used in this experiment, the first trial of NO treatment on human RCI muscle *in vitro* or *in vivo* whereas those from control muscle were not.

Muscle function and health are exquisitely dependent on the status of innervation. Immediate loss of function follows denervation and is soon after followed by atrophy, since the level of muscle contractile activity contributes to the regulation of protein breakdown [57]. Asymmetric atrophy follows tendon retraction and muscle denervation, and incomplete denervation results in differential wasting within a muscle belly [58]. Prolonged denervation leads to both atrophy and fibrosis of muscle, as detailed for example in a study of changes in denervated rat gastrocnemius muscle including increased atrogin-1, MuRF1, and fibroblast numbers, and a decrease in capillary density per fiber [59]. The same report found a correlation between the time to reinnervation and the functional outcome of the muscle [59]. Therefore, we investigated whether denervation (separate from muscle disuse after injury) was present and could contribute to the SS muscle atrophy in RCI, as significant denervation could indicate a poor prognosis.

AchR-subunit changes are used as an index of innervation state in adult muscle, since the γ-AchR subunit is expressed at very low levels in innervated adult muscle, and increases in models of denervation in rats [60] and in muscle affected by neurogenic disorders in humans [61]. Results from phase-1 [22] and phase-2 participants both revealed a strong tendency for SS to have a higher γ:ε-AchR subunit ratio than control muscle (p = 0.08 in phase-2). Moreover, expression of the γ-AchR subunit protein itself (standardized to β-actin) was three-fold higher in SS than control muscle. Given that biopsies sample only a small portion of SS and control muscles, the increase in γ-AchR protein in SS muscle is notable. Results, therefore, support the notion that the SS muscles were at least partly denervated.

Results further suggest denervation likely contributes to RCI pathophysiology, since an increase in γ-AchR is highly consistent with the atrophy of muscle fibers and the increase in atrogin-1 levels observed in SS in this study. It is not known whether denervated fibers would be homogeneously distributed within the SS or whether the condition was involved in causing RCI. However, the path of the supraspinatus nerve, deep to muscles on the scapula as it navigates through the scapular notch and around the root of the scapular spine, is thought to make the nerve susceptible to injury. Finding a major tear in the SS tendon may thus be more than diagnostic of RCI; it may indicate the value of investigating the innervation status of SS before developing a clinical plan for RCI treatment, given the relationship between muscle innervation and functional outcome. Changes in vascular density are also implicated in the impact of muscle denervation. Increases in resting blood flow and capillary bed volume are reported in denervated muscle(8;35) and vascular density was noted to be decreased after 3 and 7 months of denervation [36, 38]. In the current study, SS vascular density (detailed in a previous report,[41]) explained 9% of the variation in the amount of γ-AchR subunit protein (including all participants, regardless of the time-course of the RCI pathology). While the stiffness of fibrosis would stabilize atrophic muscle and may protect non-functional muscles from further injury [36], fibrosis can also increase susceptibility to contraction-induced injury[62] and may impede vascular perfusion, new vessel formation, innervation of regenerating fibers, and the responsiveness of SC to activation by mechanical activity [63–66].

Since the axon-guidance molecule, Sema3A, is secreted by activated SCs after their early differentiation [25, 45, 67], an alteration of SC-derived Sema3A with age, injury, or inflammation in humans may be related to innervation status and the potential for successful surgical repair of RCI. This assay for SC Sema3A was designed to collect information on changes in Sema3A in response to NO-induced activation by ISDN in culture. There was insufficient tissue in the explant cultures to allow both western blotting and sectioning for the SC-activation study.

The areal density of Sema3A staining in SCs was higher in SS muscle than in the control. Considering the atrophic, denervated state of injured SS muscle, this confirmation that the neuro-chemorepellent was secreted, is not surprising. The finding of greater Sema3A protein localization in SS than control muscle is consistent with the observed increase in γ-AchR in SS muscle through western blot experiments, indicative of SS denervation, since SCs produce the neuro-chemorepellent; this secretion of Sema3A from SCs in early differentiation is proposed as a means of preventing innervation of very new fibers, synchronizing fiber differentiation with the process of innervation [24, 25]. The present results are also consistent with previous findings in animal models of injury, including crush and toxic injection, that report increases in Sema3A following both mechanisms of injury [23, 45]. The absence of a Sema3A response to ISDN in both SS and deltoid was interesting and unexpected, given that the proportion of BrdU+/Pax7+ SCs rose in the same samples of SS cultured with ISDN. Two factors may have precluded finding such a change in this areal-density assay: neither the longevity of ISDN effects in the cultures or the time-course of Sema3A localization within SCs over a 40-hr experiment are known. This first report of Sema3A in human RCI muscle adds to a growing interest in Sema3A and could provide valuable insight into novel strategies to combat denervation-atrophy often observed with aging.

PCA results prompt many considerations, since principal components influence the overall dataset. However, interpretation of these PCs is complicated while being intriguing. As a descriptive technique, PCA does not provide a p-value and is not based on a hypothesis, so while PCA alone is not sufficient to provide information on statistical significance, it is useful for developing hypotheses for future study, and in characterizing major sources of variation in a complex dataset. Although mathematically extracted PCs may not hold significant biological meaning, the relationships prompt serious consideration of previously unexplored associations [44] as shown in a PCA study to identify age-related differences in flexion-extension gait patterns [68].

There were similarities in the way variables clustered in the PCs extracted from the PCAs. In all three analyses, the first component was related to muscle atrophy, and atrophy indicators grouped with SC activity in two of the three PCAs, indicating it is informative to retain variables on muscle atrophy in studying RCI. The second PC in all three analyses related body size (weight or height) with fibrosis, suggesting the value of these variables in future research. While the third PC was more variable across the three PCAs, the time since injury accounted for a substantial amount of total variation in the dataset. Interestingly, in the analysis with muscle as a variable, “muscle” is only extracted in the third PC, suggesting that differences in the other variables are not primarily attributable to differences in the muscle as much as to differences among individual participants. This provides valuable insight into the state of the muscle and other variables, and increases confidence in the use of deltoid as a control muscle.

The variables extracted into PCs indicate that future work should particularly investigate other aspects of muscle atrophy including denervation, and protein degradation and tissue remodeling while controlling for features such as body size and the time between symptom onset or injury and surgery. If augmented with follow-up information on post-surgical joint function in a future study, present findings would also be useful in clinical applications, such as predicting the likelihood of successful recovery after surgery. The current analysis suggests body size could be used to predict the potential for SS denervation or reduced perfusion of SS, with ensuing reduction in the chance of recovery.

Several variables may have introduced variance in the dataset, including: the variability of sampling across participants (e.g., in positioning biopsies); the possibility of focal vs. generalized damage in a biopsy; and non-homogeneous deposition of fat and collagen. These variables would have prevented finding additional differences between SS and control muscles that were not explained in PCAs. It is also important to note that in rotator-cuff muscles, morphology can differ after chronic tendon tears and neuropathy-related denervation(48). This study only captured a portrait of muscles at one moment in time, intraoperatively, and did not account for the evolution of RCI pathophysiology. A longer study of participants in the time-course from symptom onset, through surgery, and into follow-up (including a repeat MRI) would be able to evaluate whether the outcome of RCI treatment by surgery or other means, could be predicted by assessments of measures including innervation status and SC responsiveness.

The study was strengthened by confirming intraoperatively that there was a tendon tear (one participant without a tendon tear was excluded from the study in the operating room). PCAs were conducted with the aim of integrating the many findings, and suggested that innervation status, SC activity, and expression of atrogin-1 and mmp9 as indices of the active protein degradation during atrophy will be valuable in future investigations and for developing the most effective plan for treating each individual that presents with RCI. The assay of mmp9 protein was novel for RCI studies and suggests the value of studying other proteins involved in regulating collagen deposition, remodeling, and fibrosis in RCI.

## Conclusion

With an aging and less active population, debilitating conditions such as RCI will become even more common, because the prevalence of injury increases with age [5, 69, 70]. RCI produces painful symptoms and makes many activities of daily living difficult, if not impossible, to perform [1]. Furthermore, detrimental changes in muscle associated with denervation, including atrophy and fibrosis, may explain the progressive loss of function after an acute injury and also the high rate of surgical failure. A combination of atrophy and denervation, whatever the root cause, will certainly contribute to joint instability and pain. As a result, both atrophy and denervation need to be considered in therapeutic strategies for improving functional recovery following RCI. The independent confirmation that ISDN activated SCs from the supraspinatus muscle provides strong support for using a NO-donor drug, possibly in combination with mechanical stretching (e.g., by exercise), to promote muscle growth. Finally, the activity of SCs and their potential role in the process of muscle reinnervation (via Sema3A) is a relatively new field of interest [24, 25]. It was interesting to examine this phenomenon in denervated, injured, human SS muscle and find significantly more Sema3A protein localized to SCs in SS compared to control. Any potential for application to fiber reinnervation in RCI will only be available by further understanding of Sema3A biology in regenerating muscle. PCA studies suggested that assessment of indices such as SC responsiveness to activation, atrogin-1, fiber atrophy, fibrosis, and innervation status might predict the outcome of RCI surgery.
